# A Simple Electron-Density Based Force Field Model for High-Energy Interactions between Atoms and Molecules

**DOI:** 10.1021/acs.jpca.3c06724

**Published:** 2024-02-06

**Authors:** José Romero, Paulo Limão-Vieira, Kersti Hermansson, Michael Probst

**Affiliations:** † Institute of Ion Physics and Applied Physics, University of Innsbruck, Technikerstraße 25, Innsbruck 6020, Austria; ‡ Atomic and Molecular Collisions Laboratory, CEFITEC, Department of Physics, Universidade NOVA de Lisboa, Caparica 2829-516, Portugal; § Department of Chemistry-Ångström, Uppsala University, Uppsala SE-75121, Sweden; ∥ School of Molecular Science and Engineering, Vidyasirimedhi Institute of Science and Technology, Rayong 21210, Thailand

## Abstract

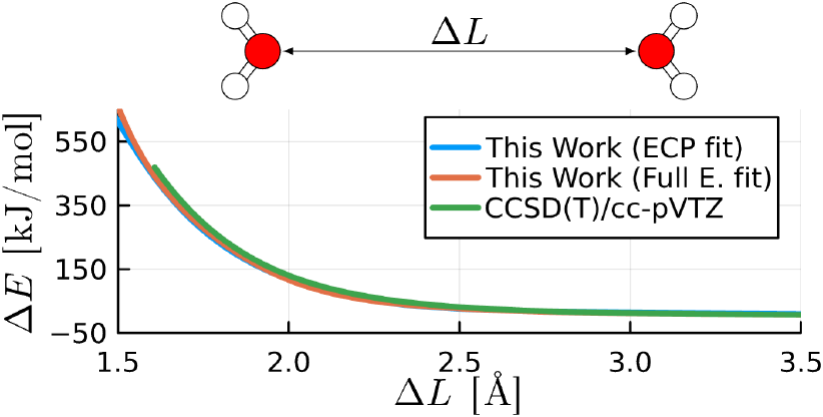

In high-energy molecular dynamics or Monte Carlo simulations, standard force fields optimized for simulations at ambient temperatures are inadequate. This is largely because their repulsive parts have been regarded as not very significant, even well below zero interaction energies. It is, therefore, not obvious which force fields to resort to for simulating hot gases or plasmas. A force field model that uses the electronic densities of noninteracting atoms or molecules within the pair approximation is introduced. We start by deriving a naïve model that neglects any exchange and correlation effects between the electronic clouds and then correct this model by adding a term calibrated from *ab initio* calculations using the CCSD(T)/cc-pVTZ level of theory. The resulting expression for this term can be regarded as a simple exchange–correlation function. We compare the results for the repulsive part of the potential energy hypersurfaces with the force fields commonly used on some dimers of small molecules.

## Introduction

For more than 50 years, a diverse array of force field models has emerged to compute interaction energies between molecules, not least with the aim of circumventing computationally demanding quantum chemical methods, such as *ab initio*, DFT, or semiempirical calculations, which strive to approximate the many-body Schrödinger equation. For nonreactive systems, these force fields typically comprise four key components:

**Bond stretching and contractions:** This involves a summation of harmonic oscillator terms to describe the stretching and contractions of bonds within a molecule.
**Bond bending:** Another summation of harmonic oscillator terms is employed to capture the bending of bonds in the molecular structure.
**Rotations of dihedral angles:** A summation is utilized to describe the rotations of dihedral angles within the molecule.
**Coulomb energies and nonbonding interactions:** This component involves a summation of Coulomb energies and other long-range interactions. It is often expressed as a combination of partial charges and a Lennard–Jones-styled potential between all pairs of atoms that do not share any of the characteristics outlined in the first three components.

Utilizing such force field models enables large-scale calculations in Molecular Dynamics (MD), employing classical physics to solve Newton’s equations of motion. This approach proves indispensable, especially when considering the computational limitations associated with more fundamental quantum mechanics-derived methods. Prominent examples of these force fields include OPLS,^1^ CHARMM,^2^ UFF,^3^ AMBER,^4^ MMFF94,^5−9^ among numerous others. The parametrization strategy of force field models varies; for instance, MMFF94 combines experimental and computational data from the Cambridge Structural Database (CSD) and MP2/6-31G* calculations.^10−24^ Explorations into alternatives for the *r*^–12^ term in the Lennard–Jones-type potential have been made such as replacing it with an  term (Buckingham potential).^25^

An important part of the quality of a force field model is the transferability. The interchangeability of the parameters of the atom classes for different molecules should be easy and/or accurate, since expecting nonexperts to design new accurate force field models for arbitrary molecules is an unrealistic expectation. Using quantum mechanical calculations to improve transferability and other features of the models is still an ongoing topic of research.^26^

In the pursuit of creating a universal molecular force field model, our approach involves deriving two-body interaction energies between molecules solely from their electronic densities. Initially, we make a simple assumption by considering the electronic densities of all molecules in the system as being independent of each other. Subsequently, we refine this assumption by introducing an exchange and correlation function based on the separated densities. This correction is inspired by the foundational concepts put forth by Hohenberg and Kohn,^27^ aiming to universally rectify the inaccuracies inherent in this naïve assumption.

Formulating the problem for a simple system involving just two atoms, where their electronic densities are represented by some linear combination of *s*-type Gaussian functions, can be solved by using exact expressions. Prior studies have demonstrated that expanding electronic densities with primitive Hermite Gaussian functions can effectively capture Coulomb and exchange–repulsion energies between interacting molecules. This concept is employed in the Gaussian Electrostatic Model (GEM) by Cisneros et al.^28−30^ Other molecular force fields developed from first principles, utilizing parameters derived from accurate *ab initio* data, include MB-Pol^31−35^ and the Effective Fragment Potential (EFP) method,^36−43^ with the latter also being an electron density-based force field.

This study introduces a comprehensive formula for incorporating terms up to the *K*^th^ order approximation for exchange and correlation correction. This is achieved through successive applications of the Laplace operator, resulting in a formula that eschews the use of inverse power law terms. Instead, it relies on linear combinations of expressions that function as noninteracting electron density functionals. In our preliminary numerical tests, this approach has demonstrated superior accuracy compared with several commonly used force field models, particularly in high-energy interactions when referencing *ab initio* calculated results. Such high energy interactions are important when surfaces are subjeted to hot particles, like in plasma-wall modeling but can also occur in the liquid state when unfavorable orientations between molecules are forced by confined spaces or other constraints.

The Methods section outlines how we model the electronic density of each molecule using data derived from quantum mechanical calculations and details the calibration process for our exchange and correlation potential. In the Results section, we present a comparative analysis of our findings for different molecules against established force field models. Finally, the Conclusion section highlights the key takeaways from our study.

## Theory and Formulation

The electronic wave function of a system AB, which consists of two molecules with a total of *N* electrons and *M* nuclei, , can be calculated through the time-independent Schrödinger equation by solving the eigenvalue problem of its well-known Hamiltonian:
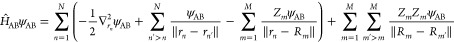
1where *r*_*n*_ and *R*_*m*_ denote the Cartesian coordinates of the electrons and nuclei respectively, *Z*_*m*_ the atomic numbers of the atoms, and  is the Hamiltonian acting on the combined system. The wave functions of the two noninteracting molecules are  and , where *N*_*A*_ and *N*_*B*_ are the number of electrons in each molecule (). The respective noninteracting Hamiltonian operators are  and .

In the so-called supermolecular approach, the interaction energy between A and B is defined as the difference in the total energy of the bonded system and the total energies of the separated molecules. In other words:
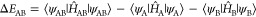
2

We now derive an expression of this interaction energy for a naïve model, which rests on the assumption that the electrons of each molecule are independent of any electrons that do not belong to the same molecule. That means that the wave function of the combined system equals the product of the wave functions of the separated molecules:
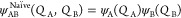
3where  and  are the coordinates of the electrons of molecules A and B, respectively. One immediate consequence of this assumption is that the electrons in the combined system are not modeled as indistinguishable particles, but rather as *semi-indistinguishable* particles (only if a pair of electrons “belong” to the same molecule they are modeled indistinguishably). The interaction energy for this naïve model is calculated via the following difference:
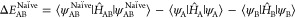
4

While  is simple to calculate if  and  are known, it lacks two properties that the exact wave function  must satisfy, namely the wave function is not (fully) antisymmetric (only interchanging the coordinates of electrons belonging to the same molecule will satisfy the antisymmetry condition, provided that  and  are exact wave functions of their respective molecules) nor is it guaranteed that  is an eigenfunction of  (in most cases it will not be). We partition this interaction energy (eq 4) into three components: Electron–electron, electron–nuclei, and nuclei–nuclei interaction energy components between electrons/nuclei that do not belong to the same molecule (,  and , respectively):
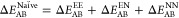
5

This naïve model can be quite appealing for systems with a modestly large number of electrons since the terms shown in eq 5 consist *only* of integrals and/or sums of the electron densities of the separated molecules and the Cartesian coordinates of the atoms, thus, making the naïve model scalable. The analytical expressions of these terms are
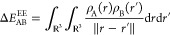
6a
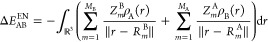
6b
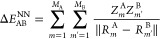
6cwhere  and  denote the Cartesian coordinates of the nuclei of the molecule, and  and  are the electron densities of the molecules A and B, respectively, when they do not interact with one another. The numbers  and  denote the number of atoms each molecule contains .

Despite its convenience, this naïve model is unlikely to provide accurate results since the naïve wave function (eq 3) is incorrect for the reasons mentioned above. To address this, we add to the naïve model an exchange and correlation interaction energy term
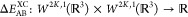
7such that
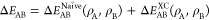
8where  denotes a Sobolev space in the usual way. We now prove that the correction term  does indeed exist by resorting to the Hohenberg–Kohn (HK) theorem. From a direct application of the HK theorem, we know that the interaction energy in eq 3 can be written in any case as the following functional:
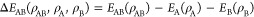
9thus, all we are left to do is to show that  and  can be mapped to . Estimating  by simply knowing  and  is likely to be problematic in many cases, especially in cases where two atoms of different molecules are placed at short distances of each other. Since the HK theorem tells us that the external potentials of  and  are functionals of  and , respectively, it is possible to map the two electron densities to a set with the Cartesian coordinates and atomic numbers of the atoms of both molecules, which is all information required to fully describe the Hamilton operator  of two molecules interacting with each other (eq 1).

In essence, this means that it is possible to map the electronic densities of the separated molecules to the interaction energy, as from the HK theorem it follows that the maps ρ_A_ 
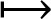
 *E*_A_ and ρ_A_ 
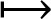
 *E*_B_ exist. Since  and  can be mapped to their respective external potentials, they can also be mapped to the Cartesian coordinates and atomic numbers. Therefore, the pair can also be mapped to the Hamiltonian operator of the interacting system:
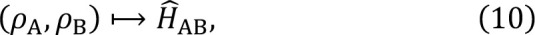
10Thus, it is also possible to map the pair of electronic densities to the ground state energy of the interacting system (the smallest eigenvalue of ), that is,
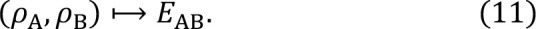
11

While we have shown that it is possible to map the two noninteracting molecular electron densities to the energy of the interacting system (eq 9), the same cannot be stated for mapping the noninteracting densities to the electronic density of the interacting system. The reason is that the map (ρ_A_, ρ_B_) 
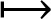
 ρ_AB_ does not exist in the case of degeneracies with more than one ground state of the interacting system (). Furthermore, using the same argument but for the separated molecules instead, it also follows that (globally) mapping the electronic density of the interacting system  to the electronic densities of the separated molecules  and  (or in other words ρ_AB_ 
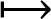
 (ρ_A_, ρ_B_)) is normally not possible either since one or both of the separated molecules may also have degenerate ground states.

Thus, far we have shown that  can be written as a functional of the pair . We now try to provide an expression for it via a heuristic approach where we separate  into electron–electron and electron–nuclei interaction components. In each of the terms, we use a  order approximation requiring the fitting of  parameters with respect to reliable experimental data, accurate (yet computationally demanding) *ab initio* calculations or a combination of both. The parameters are the weights of different functionals of the electronic densities of the noninteracting molecules.  is then defined as
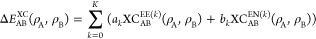
12with  and  being the optimized parameters and:
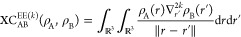
13a
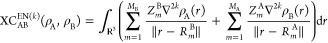
13b

The operator  used in eq 13a and 13b is shorthand notation for applying the Laplace operator  consecutive times, thus, if  the operand is unchanged. Restricting each term of our functional in eq 13a to even numbered derivatives stemming from consecutive applications of the Laplace operator guarantees (as can be seen from integration by parts) that interchanging the labels of  and  does not affect the result of the integral, that is,
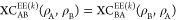
14which is a necessary condition for our model since interchanging the molecules must not alter the interaction energy (eq 2).

## Computational Methods

The electronic densities of elements within the first three rows of the periodic table are computed using density functional theory (DFT) and the B3LYP functional.^44,45^ The calculations incorporate the empirical dispersion formula GD3^46^ and an Effective Core Potential (ECP) basis-set denoted as CEP-31G.^47−49^ The electronic densities obtained from these calculations are then approximated using three s-type Gaussian functions for the ECP basis-set, and nine s-type Gaussian functions for the full electron basis-set aug-cc-pVTZ,^50^ both at the same level of theory.

To account for charge redistribution in isolated monomers, Mulliken charges^51^ are employed. This compensation is essential for mitigating charge redistribution effects when monomers come into close contact. Notably, this model is nonpolarizable, it does not consider charge redistribution between molecules.

In ECP calculations, effective atomic numbers replace actual atomic numbers to appropriately adjust for the reduced number of electrons due to the implementation of Effective Core Potentials. Further details, including the methodology for compensating for charge redistribution, can be found in the Supporting Information.

Representing the electronic densities of isolated molecules as linear combinations of s-type Gaussian basis functions, whether atom-centered or not, facilitates the derivation of exact closed-form solutions for the integrals presented in eqs 6a, 6b, 13a, and 13b. The exact expressions for these terms, up to order 2, are provided in the Supporting Information. This approach simplifies the computation of integrals, offering precise solutions for the given equations. The Supporting Information contains more comprehensive details and expressions.

The exchange and correlation functional described in eq 12 is fine-tuned against Coupled Cluster Singles and Doubles (CCSD) calculations,^52−55^ including triple excitations (CCSD(T)),^55,56^ specifically for water dimers in different conformations. To evaluate the generalizability of this calibrated model, we applied it to predict interaction energies in neutral and anionic O_2_ dimer systems, with reference values obtained from restricted open (RO) shell CCSD(T) calculations^57^ to prevent spin contamination. All CCSD(T) calculations throughout this manuscript utilize the all-electron basis-set cc-pVTZ.^50^

The calibration process focuses on water dimer interactions due to their prominence in both dedicated and general force fields. Additionally, the choice of H_2_O is advantageous because of its small size, allowing the utilization of high-accuracy *ab initio* methods. This convenience facilitates the generation of an ample amount of reference data necessary for fine tuning and calibrating our model. The water dimer, thus, serves as a valuable and practical benchmark for ensuring the accuracy and effectiveness of the calibration process.

**Figure 1:**
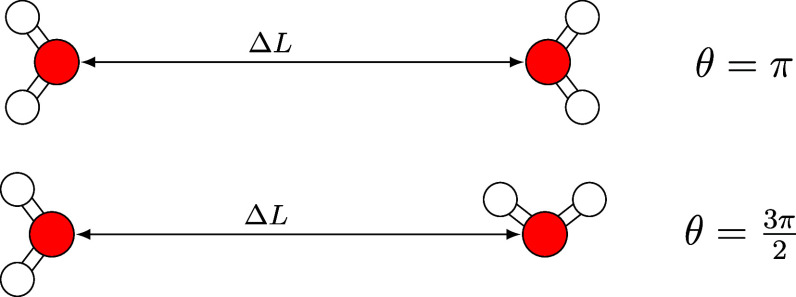
H_2_O dimers in “oxygen-bonded” configurations considered to determine the coefficients in the correction terms (eq 12).

**Figure 2:**
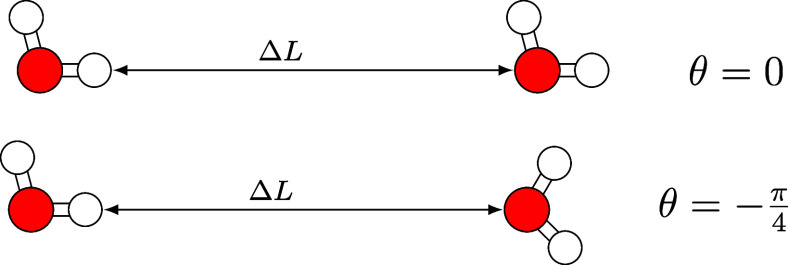
H_2_O dimers in “hydrogen-bonded” configurations considered to determine the coefficients in the correction terms (eq 12).

A total of 10 000 water dimers are used to calibrate the XC function. We separated them into what could be called non-hydrogen-bonded and hydrogen-bonded sets of 5000 configurations each. In the first one, the O···O distance is varied, and in the second one, the intermolecular O···H distance. Figure 1 shows the start and end of the first set of configurations ,  and Figure 2 shows the same for the second (, ). The configurations are sampled in a  grid for the angular and the distance components, respectively.

**Figure 3:**
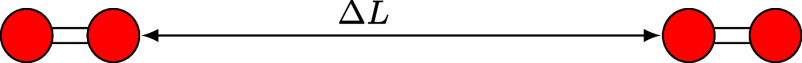
O_2_ dimer configurations.

For the CCSD(T) calculations for O_2_ dimers, the charge states neutral, cation, and dication are considered. Both molecules are placed in a colinear arrangement, and their distance is varied (). The lowest-energy spin states taken, for example, triplet states for isolated O_2_ neutral molecules), and the interatomic distance  is evaluated on a grid of 300 evenly distributed points (Figure 3).

We evaluate the interaction energies computed at the CCSD(T)/aug-cc-pVTZ level of theory across our entire calibration data set. We contrast these reference values with the results obtained from both our calibrated models, ECP and full electron. Furthermore, we compare these energies with those from widely used and frequently cited molecular force field models. Specifically, we include GAFF,^58^ MMFF94S^59^ (using the open source OpenBabel software^60^ for both) Jorgensen’s TIP3P water model,^61^ MB-Pol^31−35^ (using MBX^62^ and EFP,^36−43^ and the open-source library LIBEFP).^63^

The dependence of the accuracy on the approximation order is expressed by the Relative Absolute Errors (RAEs) of the predicted interaction energies against the CCSD(T) results on all calculations of the (H_2_O + H_2_O) complexes:
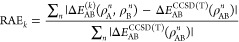
15

All the DFT and CCSD(T) calculations used in this manuscript were performed using the Gaussian 16.3 Software,^64^ using the High-Performance Computing (HPC) facility of the University of Innsbruck. A combination of a genetic algorithm and the Broyden–Fletcher–Goldfarb–Shanno (BFGS) algorithm^65−68^ was used for all the nonlinear least-squares optimizations, using Julia programming language-based scripts.

## Results and Discussion

**Figure 4:**
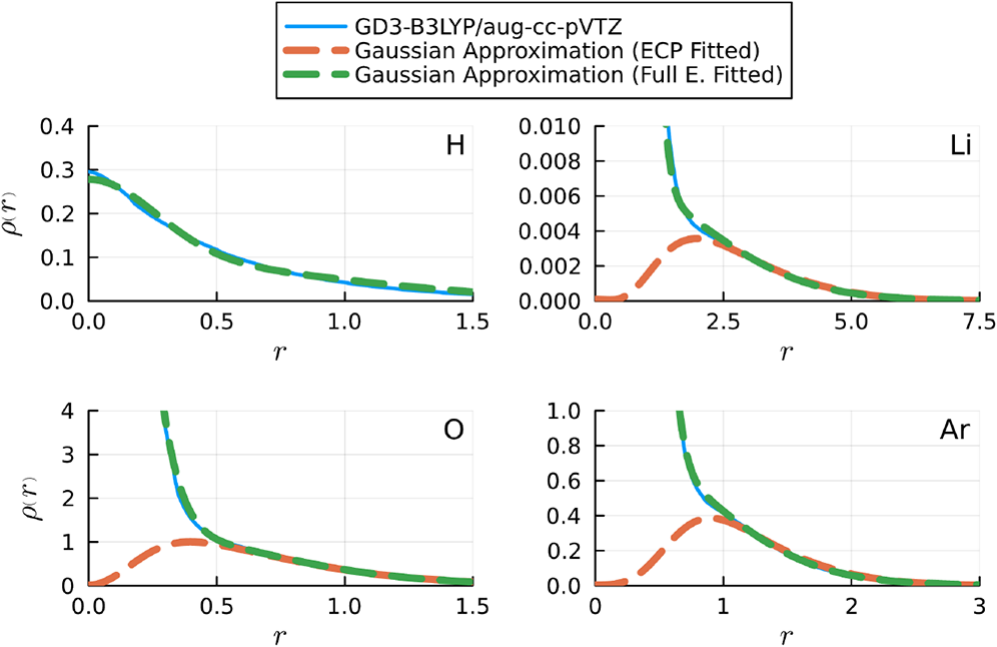
Comparison of the approximated radial component of the electronic densities (dashed lines) versus the respective DFT results (solid lines) for helium, lithium, oxygen, and argon atoms. Radial distances are given in Bohr.

The electronic densities of the elements in the first three rows of the periodic table are effectively replicated using three Gaussian functions in ECP basis-set calculations, although the approximation of the electronic densities of H and He appears coarser than that for the other atoms, as evidenced in Figure 4. This discrepancy is expected, given that the full electron density for these two elements comprises the summation of two 1s-type orbitals, dictated by the chosen basis set. Fortunately, this issue is not persistent for other elements, as their 2s atomic orbitals inherently exhibit smoother characteristics.

The coefficients of the Gaussians for these elements are listed in the Supporting Information. Conversely, when employing a full electron basis-set, accurate reproduction of electronic densities is achieved through the utilization of nine s-type Gaussian functions for all atoms in the first three rows. It is worth noting that in contrast to the ECP scenario, the expressions fitted from full electron calculations are less accurate for very small distances to the nucleus.

**Figure 5:**
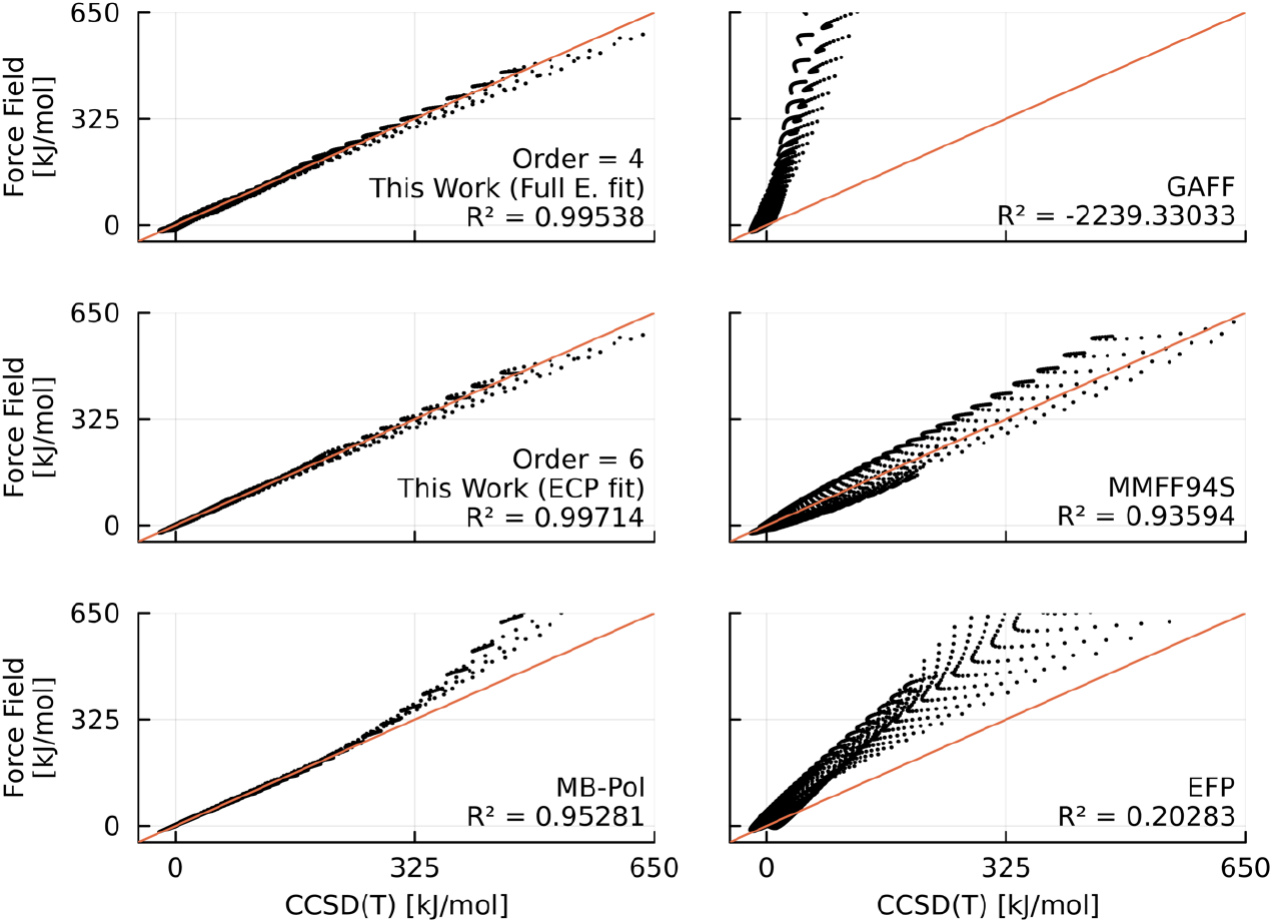
Comparison between the interaction energies obtained from the CCSD(T)/cc-pVTZ level of theory (singlets) against the results from our corrected models and four established force field models. The data points displayed here are the same used to fit the parameter of our model.

Our corrected models of order six (ECP fitted) and four (full electron fitted) demonstrate commendable accuracy in replicating *ab initio* data for energies, surpassing even established force fields, as illustrated in Figure 5. It is important to note that this comparison holds some subjectivity, primarily due to the heightened emphasis on configurations where the interaction between the water molecules in the dimers is markedly repulsive. Our objective is to cultivate a model capable of accurately reproducing such regions, as they bear significance in simulations of systems subjected to high temperatures or large atomic kinetic energies in general.

In a broader context, the MMFF94S, EFP and MB-Pol models exhibit proficiency in maintaining accuracy at elevated interaction energies, with the latter emerging as the superior choice overall. However, even though MB-Pol generally outperforms our ECP and full electron models at lower energy interactions, it tends to overshoot at higher energies. This is expected, considering that MB-Pol employs inverse power laws for determining interaction energies. Another pivotal distinction lies in the polarizable nature of MB-Pol, necessitating an additional computational step at runtime, a feature absent in our nonpolarizable model. It is essential to recognize these nuances when considering the trade-offs between accuracy and computational efficiency in selecting an appropriate model for simulations.

**Figure 6:**
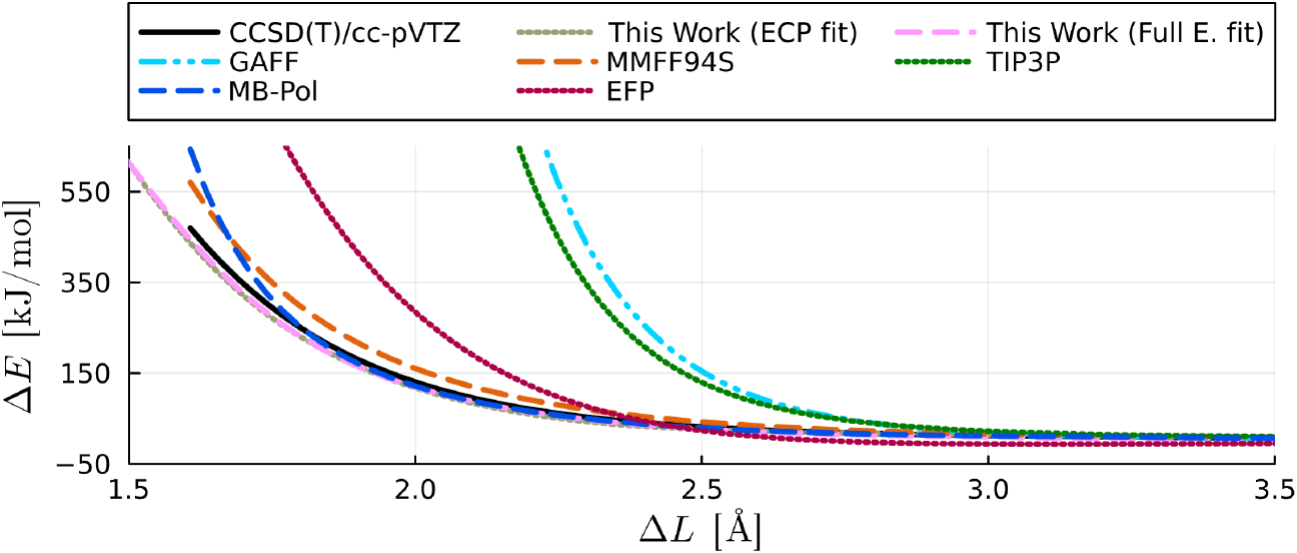
Interaction energy of a water dimer in the O···O reaction coordinate shown in Figure 1 () obtained from *ab initio* calculations (singlets) using CCSD(T)/cc-pVTZ level of theory and several force field models including ours with an order six and four corrections for the ECP and full electron models, respectively. The curves of our “ECP fit” and “full electron fit” models overlap with each other.

We also find that for interatomic distances shorter than 3Å in the repulsive O···O reaction coordinate of the water dimer, both GAFF, TIP3P, and EFP exhibit shortcomings in reproducing the interaction energy, as depicted in Figure 6. In contrast, our ECP and full electron calibrated models, along with MMFF94S and MB-Pol, maintain accuracy even when interatomic distances are shorter than 2 Å. For energies below 150 kJ/mol, MB-Pol shows remarkable accuracy, outperforming both the ECP and full electron calibrated models. However, for higher energy configurations, where shorter interatomic distances appear, MB-Pol also overestimates the energies, whilst both the ECP and full electron calibrated models remain accurate. This observation highlights the strengths of our calibrated models in accurately capturing interaction energies, especially in regions with challenging interatomic distances.

**Figure 7:**
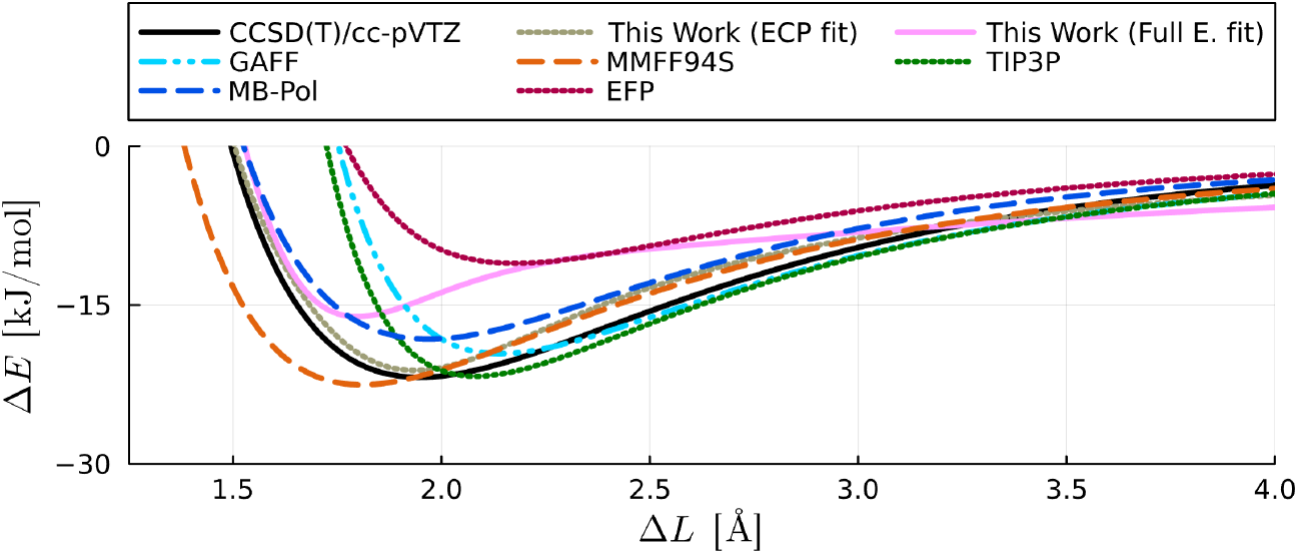
Interaction energy of a water dimer in the O···H reaction coordinate shown in Figure 2 () obtained from *ab initio* calculations (singlets) using CCSD(T)/cc-pVTZ level of theory and several force field models including ours with an order six and four corrections for the ECP and full electron models, respectively.

An analogous inspection of hydrogen-bonded configurations in Figure 7 yields a slightly different perspective. All models except EFP demonstrate accuracy in predicting attractive regions. However, in the plotted region, only our ECP and full electron calibrated models, along with MB-Pol, accurately modeled the repulsive part. This again underscores the effectiveness of our calibrated models and of MB-Pol in capturing both attractive and repulsive interactions in hydrogen-bonded configurations.

**Figure 8:**
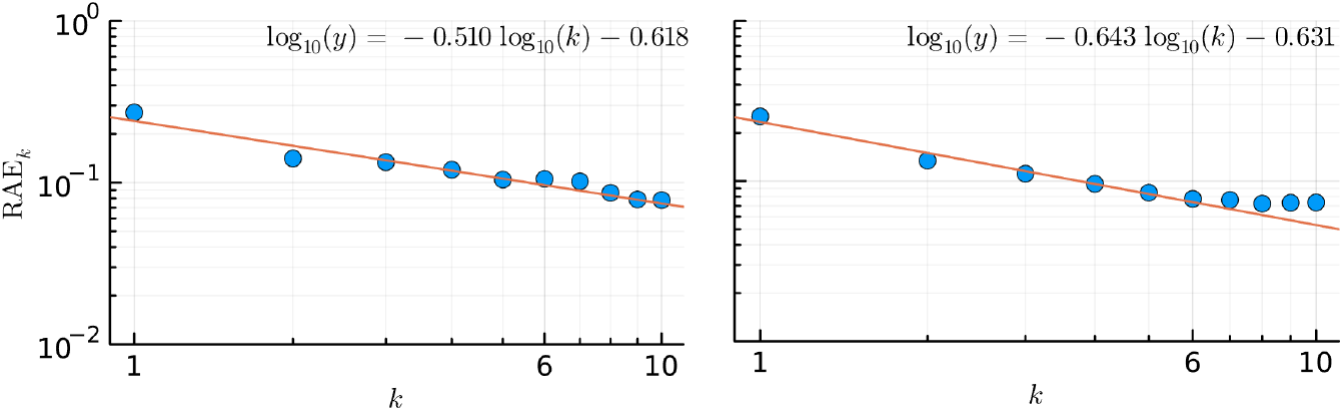
Normalized error of the interaction energies for the full electron model (left panel) and ECP model (right panel) of all the configurations shown in Figure 5 as functions of the approximation order (labeled as ). The red line is a linear fit through the data points.

Figure 8 illustrates the decay of the Relative Absolute Error (RAE), as defined in eq 15, on a double logarithmic scale. For the sixth order, the RAE stands at approximately 0.05 for the ECP fitted model. However, as orders exceed 6, the improvement in the ECP model performance decelerates. The orange line in the figure is a fit to the RAE values for orders 1 to 6, and its slope and offset are depicted in the inset. Despite the ECP model with orders beyond six exhibiting a smaller total error, indications of overfitting start emerging beyond the sixth order. Small artifacts become noticeable near the minimum potential well of the same O···H reaction coordinate depicted in Figure 7.

Conversely, the error of our full electron model diminishes linearly on the double logarithmic scale, as shown in Figure 8. However, for orders surpassing four, unphysical oscillations manifest in the O···H reaction coordinate, as shown in Figure 7. To precisely extrapolate interaction energies along this reaction coordinate, expanding our calibration data set to include water dimer configurations with even shorter interatomic distances is imperative.

This limitation is anticipated when scrutinizing the proposed exchange and calibration functional, as depicted in Figure S1 of the Supporting Information. The higher the order of each functional in the sum, the more oscillations occur, presenting challenges in extrapolating interaction energies for specific reaction coordinates that may or may not be part of the calibration data set.

**Figure 9:**
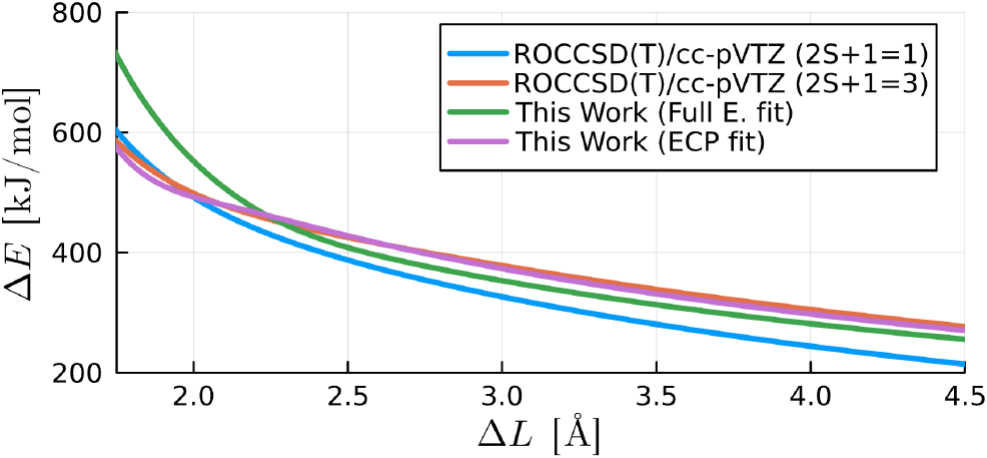
Interaction energy of an  complex in the reaction coordinate shown in Figure 3 obtained from CCSD(T)/cc-pVTZ level of theory and our calibrated model from neutral water dimer calculations (same level of theory) using an order six and four corrections for the ECP and full electron models, respectively. The red and gray curves practically overlap in the plot.

Concerning the O_2_ dimer systems, both our ECP and full electron calibrated models demonstrate the ability to predict interaction energies for an  complex in the triplet spin state, as illustrated in Figure 9. Remarkably, despite being exclusively calibrated from neutral water dimer calculations, these models perform well for this different molecular configuration. However, for interatomic distances shorter than approximately 2.5Å, the accuracy of our full electron model diminishes, while the ECP model maintains a fair degree of accuracy.

**Figure 10:**
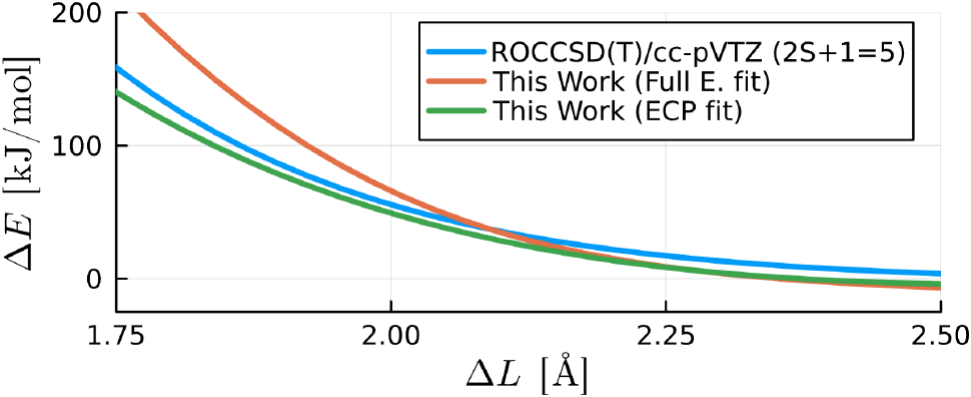
Interaction energy of an  complex in the reaction coordinate shown in Figure 3 obtained from CCSD(T)/cc-pVTZ level of theory and our calibrated model from neutral water dimer calculations (same level of theory) using an order six and four corrections for the ECP and full electron models, respectively.

A similar conclusion is drawn by examining of  complexes in an identical reaction coordinate, as depicted in Figure 10. Both models yield results identical to *ab initio* data for interatomic distances larger than 2.25Å, and beyond this point, the ECP calibrated model is notably more accurate. This evaluation highlights the versatility of our calibrated models in extending their predictive capabilities beyond the specific calibration set, while also showcasing the nuanced performance differences between the ECP and full electron models in certain molecular configurations.

### Deficiencies due to the Limited Data Base and/or Lack of Polarizability

**Figure 11:**
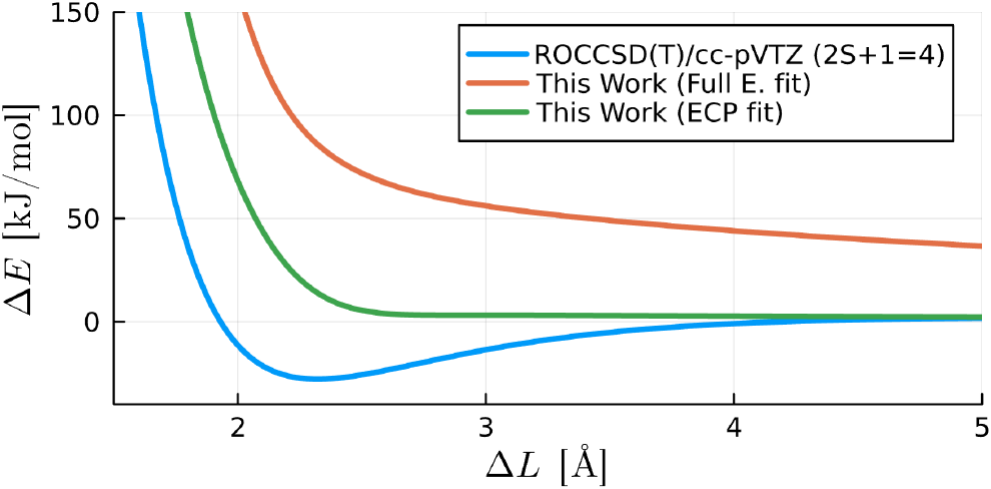
Interaction energy of an  complex in the O···O reaction coordinate shown in Figure 3 obtained from CCSD(T)/cc-pVTZ level of theory and our calibrated model from neutral water dimer calculations (same level of theory) using an order six and four corrections for the ECP and full electron models, respectively.

While our model has proven successful in replicating potential energy surfaces between monomers where no covalent bonds are formed or broken, it struggles when modeling the interaction energies within an  complex along the reaction coordinate depicted in Figure 11. Both our “ECP fit” and “full electron fit” models predict an entirely incorrect repulsive potential, in contrast with the attractive potential observed in the *ab initio* reference data. Notably, the full electron model exhibits a noticeably lower level of accuracy in this context.

Determining the exact reasons for these inaccuracies is challenging. The absence of reference data for interactions involving both neutral and anion species of the same molecule will play a role. Additionally, the methodology of modeling anion electron densities by simply scaling them to match the electron populations of an anion monomer might be insufficient for accurately simulating interactions with anions. Further investigation and refinement of the model in the context of anionic species are warranted to enhance its accuracy and reliability in such scenarios.

### Deficiencies of the Exchange and Correlation Functional

**Figure 12:**
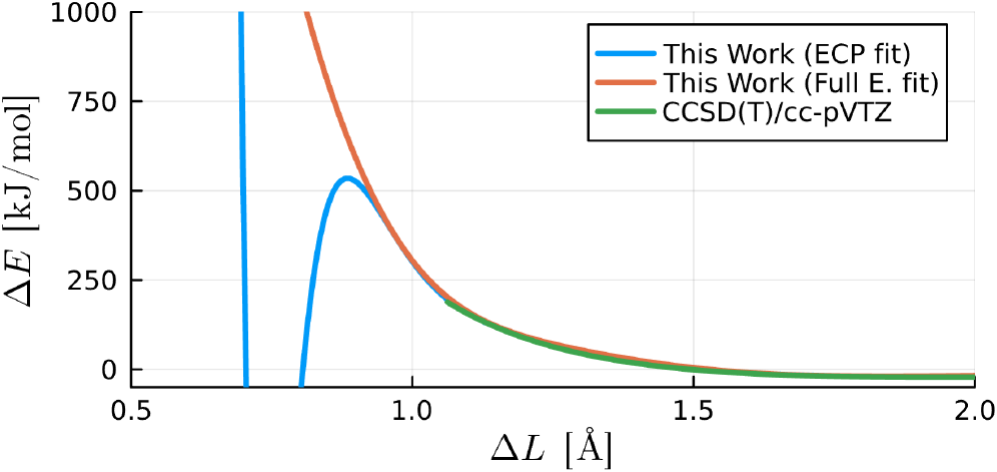
Interaction energy of a water dimer in the O···H reaction coordinate shown in Figure 2 () obtained from CCSD(T)/cc-pVTZ calculations and our order six and four ECP and full electron models, respectively. The interaction energies are extrapolated to shorter interatomic distances with our model.

In examining the repulsive region of the O···H reaction coordinate of the water dimers, our ECP fitted model remains accurate up to about 250 kJ/mol, whereas our full electron model maintains accuracy up to 2000 kJ/mol (trimmed to 1000 kJ/mol in Figure 12 for illustrative purposes) in this reaction coordinate. As interatomic distances decrease, such as in the intermolecular , our model encounters a point where strong, unphysical oscillations become apparent (Figure 12). Notably, for the ECP basis-set, these oscillations occur at much shorter interatomic distances.

While oscillations are also observed for our fitted model with the full electron basis-set, the precise reason is not entirely clear. Potential contributing factors include a lack of reference data with short interatomic distances, the oscillatory nature of our proposed exchange and correlation functionals at higher orders (as seen in Figure S1 of the Supporting Information), the truncation of our exchange and correlation functional at a finite order, lack of enough reference data in high interaction energy regions, and/or the lack of polarizability in our model. Nevertheless, both our ECP and full electron fitted models accurately extend to largely repulsive regions, with the full electron model being the preferred choice for simulations with more extreme energy requirements.

## Conclusions

In our study, we present a comprehensive framework for modeling intermolecular interaction energies from electron densities by using basis functions designed for atoms in the first three rows of the periodic table. We employ both ECP and full electron calculations with three and nine s-type Gaussian functions, respectively. Our approach initiates with a naive model, assuming the independent movement of electrons in each molecular clouds. A more accurate prediction is attained through a series expansion involving consecutive applications of the differential Laplace operator, interpreted as exchange and correlation interaction correction terms. Leveraging the Hohenberg–Kohn theorem, we demonstrate the existence of such a functional, requiring a relatively high-order expansion (6 or higher) for the accurate modeling of interaction energies. However, challenges arise in high-order expansions for extrapolating to extremely high interaction energy regions due to the oscillatory nature of our proposed exchange and correlation functional.

Testing our force field model, including both ECP and full electron versions, on a variety of water and oxygen dimer configurations yields accurate predictions for interaction energies within 400 kJ/mol for the ECP case, while the full electron model displays the capability for much higher energies. Notably, our model outperforms some popular force fields in the literature, particularly in the repulsive “high-temperature” part of potential energy surfaces, especially for interaction energies below 400 kJ/mol.

While our model demonstrates efficiency, being on average 10 times slower than TIP3P for water dimer interaction energies, it remains considerably faster than the *ab initio* methods. Challenges arise in predicting interaction energies for the O_2_ dimer anion, indicating that the expansion coefficients obtained at the present state of our models are not universal, emphasizing the need for careful testing. For molecular dynamics simulations requiring accurate modeling of high-energy regions of potential walls, such as in hot plasmas, the model calibrated from full electron calculations is better suited. Notably, it did not display signs of unphysical oscillations in the few high-energy extrapolation cases studied (for an order of four expansion). Conversely, our ECP-fitted model with an order six expansion proves to be superior to the full electron-fitted model for lower-energy simulations, such as the ones relevant in biophysics or drug discovery.

## Data Availability

All the data and programming scripts used to write this manuscript are freely available under a GPL-3.0 license, and can be downloaded from the GitHub repository: https://github.com/JoseRodriguezRomero/ElectronDensityForceModel

## Supporting Information Available

Tables with all the fitted coefficients of the s-type Gaussian functions against ECP and full electron DFT calculated electron densities, exact expressions for the exchange and correlation functionals up to order two and their recursive expressions for higher order functionals, and the exchange and correlation coefficients for an order four full electron and an order six ECP models obtained from least-squares minimization against all the *ab initio* water dimer calculations (PDF)
