# Supplementary material for: A Simple Electron-Density Based Force Field Model for High-Energy Interactions between Atoms and Molecules
Source: J Phys Chem A. 2024 Feb 6;128(6):1163–72. doi: 10.1021/acs.jpca.3c06724 (PMC10875675; doi:10.1021/acs.jpca.3c06724)
Supplement: Supplementary file 1 — jp3c06724_si_001.pdf [file jp3c06724_si_001.pdf]

Supplementary Material: A Simple Electron-Density Based Force Field Model for High-Energy Interactions between Atoms and Molecules

José Romero,[1,2] Paulo Limão-Vieira,[2] Kersti Hermansson,[3] and Michael Probst[*,1,4]

[1] Institute of Ion Physics and Applied Physics, University of Innsbruck, Technikerstraße 25, 6020 Innsbruck, Austria.

[2] Atomic and Molecular Collisions Laboratory, CEFITEC, Department of Physics, Universidade NOVA de Lisboa, 2829-516 Caparica, Portugal.

[3] Department of Chemistry, Ångström Laboratory, Uppsala University, Uppsala 751 21, Sweden

[4] School of Molecular Science and Engineering, Vidyasirimedhi Institute of Science and Technology, Rayong 21210, Thailand.

*michael.probst@uibk.ac.at

Approximation of the Electronic Densities

The electronic density of any molecule is approximated as a linear combination of gaussian functions centered in the atoms, that is:

$$\rho_e(r) = \sum_{n=1}^{N} \sum_{k=1}^{3} c_{n,k} \frac{Z_n^{\mathrm{Eff}} - \varrho_n}{Z_n^{\mathrm{Eff}}} \left(\frac{\lambda_{n,k}}{\pi}\right)^{3/2} \exp\left(-\lambda_{n,k} \|r - r_n\|^2\right), \tag{S1}$$

where the coefficients $c_{n,k}$, $\lambda_{n,k}$ and Z_n^{Eff} are element specified in Table S1, ϱ_n is the Mulliken charge of the nth atom of the molecule obtained from *ab initio* calculations for the monomer molecule in vacuum, r_n is the position of the nth of the molecule and N is the number of atoms of the molecule. Naturally for all atoms the condition $Z_n^{\mathrm{Eff}} = \sum_{k=1}^{3} c_{n,k}$ is *necessarily* true (excluding floating-point precision errors).

Table S1 Coefficients used to approximate the electronic density (averaged from line scans along the x, y and z axes between the origin and up to a distance of 25 a_0 – sum of electrons with alpha and beta spin) of each element on the first three rows of the periodic table obtained from non-linear least squares minimization against DFT results with the basis-set CEP-31G.

	$2S+1$	Z	Z^{Eff}	c	λ
H	2	1	1	0.0560384663	6.4891380598
				-3.8350294175	0.6602381433
				4.7789825290	0.6602379739
He	1	2	2	0.1154965782	20.7960680562
				-7.4980066326	1.8649595111
				9.3822136528	1.8649595136
Li	2	3	1	1.0742154658	0.0995837849
				1.6710452453	1.0335545866

				-1.7452607228	1.0167791686
Be	1	4	2	2.1191828567	0.2110703089
				1.3640078199	2.5438539255
				-1.4831909869	2.4642399930
B	2	5	3	2.7286101934	0.3034723468
				1.5220254292	1.3945941855
				-1.2506361464	1.7758340232
C	3	6	4	1.2731715309	0.2413599331
				2.8524137430	0.7090489470
				-0.1255852914	5.9730507306
N	4	7	5	1.5290249588	0.3210471862
				3.5729989560	1.0112933174
				-0.1020239143	11.2757988275
O	3	8	6	2.2322546142	0.4558507829
				3.8680898721	1.4561260735
				-0.1003449210	17.5892339938
F	2	9	7	2.9874899707	0.6252726079
				4.1153977001	2.0168889406
				-0.1028901107	25.3396865763
Ne	1	10	8	3.7964272240	0.8316425609
				4.3137870510	2.7068322521
				-0.1102230912	33.9799882925
Na	2	11	1	1.1093339714	0.0868213359
				1.6534008747	0.7252989069
				-1.7627348597	0.7077677322
Mg	1	12	2	2.2425040312	0.1477103339
				1.3011987461	1.1940647426
				-1.5437028567	1.1179254449
Al	2	13	3	3.3169451948	0.1915665690
				1.1998574162	1.7107926191
				-1.5168029427	1.5621835716
Si	3	14	4	31.8404690060	2.0594585811
				-32.2910949533	2.0484478936
				4.4506251229	0.2541638361
P	4	15	5	54.5816193530	2.5209402768
				-55.1885187244	2.5104963357
				5.6068975805	0.3300374846
S	3	16	6	68.2022557138	3.1243694200
				-68.9269226526	3.1120829865

				6.7246627841	0.4104813286
Cl	2	17	7	68.1398271228	3.8169326678
				-68.9891563371	3.7993365539
				7.8493211258	0.5026179890
Ar	1	18	8	68.0721240719	4.5129686287
				-69.0573204879	4.4891013881
				8.9851817479	0.6047157393

Table S2 Coefficients used to approximate the electronic density (averaged from line scans along the x, y and z axes between the origin and up to a distance of 25 a_0 – sum of electrons with alpha and beta spin) of each element on the first three rows of the periodic table obtained from non-linear least squares minimization against DFT results with the basis-set aug-cc-pVTZ.

	$2S+1$	Z	c	λ
H	2	1	0.0484204377	7.5979920898
			-1.6148539029	0.6812578838
			2.5664223098	0.6812578518
He	1	2	0.3306129192	9.1039001695
			0.0073241598	92.3923980909
			1.6619970957	1.3764942865
Li	2	3	-1030.1959410498	0.0902798371
			-279.2279308849	2.8716168066
			0.0276923393	640.9812908224
			-217.4576964969	0.0913685609
			735.8719735870	0.0913686786
			0.0106693275	2445.2712091313
			280.3847705688	2.8716168080
			512.8919738668	0.0892148292
			0.6944792450	11.2268655156
Be	1	4	-1029.9342080972	0.1947146745
			-279.2112840227	6.2640244868
			0.0367042397	640.9843118403
			-217.1945202552	0.1983225217
			736.1333717772	0.1983225339
			0.0108256133	2445.2682062810
			280.4016456768	6.2640244835
			513.1553960962	0.1912058331
			0.6020581977	23.6186686725
B	2	5	-1029.6835711546	0.3260793958
			-279.2172298067	10.2888592013

			0.0489262743	640.9836158304
			-216.9464914101	0.3343740541
			736.3843005780	0.3343741464
			0.0171990249	2445.2742422156
			280.3957469864	10.2888592178
			513.4029709783	0.3180373735
			0.5981334953	38.3056593709
C	3	6	-1029.4387203890	0.5080577978
			-279.2166900637	15.1576518247
			0.0543568343	640.8753205822
			-216.7004171421	0.4845890874
			736.6271569965	0.5179813657
			0.0307623085	2445.2854440620
			280.3962545793	15.1576522169
			513.6479894580	0.4845893513
			0.5992864729	57.9093314613
N	4	7	-1029.1960213565	0.7261239132
			-279.2516383972	19.5131338058
			0.0634570246	640.6633489038
			-216.4590560710	0.6878371655
			736.8683778259	0.7423964332
			0.0455087366	2445.2950407706
			280.3612937930	19.5131331757
			513.8884991872	0.6878370720
			0.6795516301	72.9202519758
O	3	8	-1028.9583004076	0.9525225739
			-279.3316986090	21.4075497552
			0.0892178012	640.3019777413
			-216.2261795406	0.8959478176
			737.1014204444	0.9767679175
			0.0589770798	2445.3090719536
			280.2812410279	21.4075484806
			514.1220647141	0.8959478363
			0.8632216037	78.1826073252
F	2	9	-1029.1100375598	0.6395420123
			-278.3063903583	2.5476442497
			0.7448766610	110.6732358768
			-216.9607617547	0.6124730824
			736.8997141143	0.6515137615

			0.0860344164	2454.1586625537
			281.3063981807	2.5476442290
			513.3735467315	0.6124634433
			0.9665916897	33.0590537265
Ne	1	10	-1028.8024503460	0.7888380083
			-278.1120656326	3.2665711251
			0.8202583595	123.1102354892
			-216.9539852535	0.8140238235
			737.1769460388	0.8140549670
			0.1060239653	2459.8363538952
			281.5008920699	3.2665695392
			513.3882512929	0.7655604755
			0.8760960396	38.8674670258
Na	2	11	-1029.6551687496	0.1218902916
			-277.6470701762	1.5331057717
			1.4902603277	83.4894010756
			-217.8109039892	0.1201893839
			736.3197614372	0.1233454037
			0.1548368325	2459.8201701452
			281.9658726229	1.5330427114
			512.5311916207	0.1190817748
			3.6511599243	4.2292556020
Mg	1	12	-1029.3777416676	0.1485649374
			-277.6181115600	2.1730232909
			1.4659313552	100.1470336163
			-217.5400152770	0.1439277042
			736.6005475758	0.1504971934
			0.1730890496	2459.7956247611
			281.9947718074	2.1730232603
			512.8029594794	0.1439302551
			3.4985253250	5.5173555385
Al	2	13	-1029.1167622445	0.1672095754
			-277.5967016835	2.9222490682
			1.4415557256	116.7055587062
			-217.2715127171	0.1718401740
			736.8621681385	0.1718365693
			0.1879392977	2459.7610149146
			282.0162784248	2.9222490584
			513.0708705188	0.1628101535

			3.4061182043	6.9425047982
Si	3	14	-1028.8390346918	0.2189948247
			-277.4382571089	3.9336649192
			1.4143885301	134.7754289517
			-216.9929246915	0.2090528720
			737.1383582927	0.2232748770
			0.2068205898	2459.6920683090
			282.1747340279	3.9342317032
			513.3480247184	0.2091163265
			2.9878393891	8.9089501274
P	4	15	-1028.5576512346	0.2837611733
			-277.1457074553	5.3672744164
			1.3808259948	155.0780927428
			-216.7106003660	0.2702032747
			737.4209564451	0.2897274230
			0.2273477574	2459.5725438743
			282.4671725224	5.3672769755
			513.6306066823	0.2702023310
			2.2869933298	11.8163348993
S	3	16	-1028.2771507209	0.3506135515
			-276.8565030332	7.0187771350
			1.3423000785	177.5658651740
			-216.4305838583	0.3321408273
			737.6998573181	0.3588275410
			0.2456820215	2459.3747039591
			282.7563746274	7.0187781815
			513.9115721154	0.3321409194
			1.6083898706	16.0065694580
Cl	2	17	-1027.9832597030	0.4317318168
			-277.2055011487	6.6001443906
			1.3207407456	198.2746531167
			-216.1457308029	0.4072142311
			737.9920886703	0.4427112359
			0.2688827168	2459.0205356813
			282.4070832926	6.6274907726
			514.1972997852	0.4072187254
			2.1483280896	16.9772062628
Ar	1	18	-1027.7057619462	0.5074554063
			-277.7176624969	13.3034378538

	1.2814161281	227.2530423460
	-215.8583551935	0.4778532303
	738.2583034107	0.5212413946
	0.2785019092	2457.7803515504
	281.8954631821	13.2041204451
	514.4359739802	0.4774217391
	3.1320506797	20.9186585799

Exchange and Correlation Functionals

If the electronic density for a molecule can be approximated as a linear combination of Gaussian functions (centered or not at each of the atoms of the molecule), then, it immediately follows that all one needs is a general formula for the exchange and correlation where just two Gaussian functions are used (one gets differentiated and the other one does not, it does not matter which of them), since the general case for a molecule will be a linear combination of these. The solutions for this simpler case up to order three are:

$$\rho_{\mathrm{A}}(r) = c_{\mathrm{A}}\left(\frac{\lambda_{\mathrm{A}}}{\pi}\right)^{3/2} \exp(-\lambda_{\mathrm{A}}\|r - r_{\mathrm{A}}\|^2) \tag{S2}$$

$$\rho_{\mathrm{B}}(r) = c_{\mathrm{B}}\left(\frac{\lambda_{\mathrm{B}}}{\pi}\right)^{3/2} \exp(-\lambda_{\mathrm{B}}\|r - r_{\mathrm{B}}\|^2) \tag{S3}$$

$$\mathrm{XC}_{\mathrm{AB}}^{\mathrm{EE}(0)}(\rho_A,\rho_B) = -\frac{c_{\mathrm{A}}c_{\mathrm{B}}\mathrm{erf}\left(\sqrt{\frac{\lambda_{\mathrm{A}}\lambda_{\mathrm{B}}}{\lambda_{\mathrm{A}}+\lambda_{\mathrm{B}}}}\|r_{\mathrm{A}} - r_{\mathrm{B}}\|\right)}{\|r_{\mathrm{A}} - r_{\mathrm{B}}\|} \tag{S4}$$

$$\mathrm{XC}_{\mathrm{AB}}^{\mathrm{EE}(1)}(\rho_{\mathrm{A}},\rho_{\mathrm{B}}) = -\frac{4c_{\mathrm{A}}c_{\mathrm{B}}}{\sqrt{\pi}}\left(\frac{\lambda_{\mathrm{A}}\lambda_{\mathrm{B}}}{\lambda_{\mathrm{A}}+\lambda_{\mathrm{B}}}\right)^{3/2} \exp\left(-\frac{\lambda_{\mathrm{A}}\lambda_{\mathrm{B}}}{\lambda_{\mathrm{A}}+\lambda_{\mathrm{B}}}\|r_{\mathrm{A}} - r_{\mathrm{B}}\|^2\right) \tag{S5}$$

$$\mathrm{XC}_{\mathrm{AB}}^{\mathrm{EE}(2)}(\rho_{\mathrm{A}},\rho_{\mathrm{B}}) = -\frac{8c_{\mathrm{A}}c_{\mathrm{B}}(\lambda_{\mathrm{A}}\lambda_{\mathrm{B}})^{5/2}(2\lambda_{\mathrm{A}}\lambda_{\mathrm{B}}\|r_{\mathrm{A}} - r_{\mathrm{B}}\|^2 - 3(\lambda_{\mathrm{A}}+\lambda_{\mathrm{B}}))}{\sqrt{\pi}(\lambda_{\mathrm{A}}+\lambda_{\mathrm{B}})^{7/2}\exp\left(\frac{\lambda_{\mathrm{A}}\lambda_{\mathrm{B}}}{\lambda_{\mathrm{A}}+\lambda_{\mathrm{B}}}\|r_{\mathrm{A}} - r_{\mathrm{B}}\|^2\right)} \tag{S6}$$

$$\mathrm{XC}_{\mathrm{AB}}^{\mathrm{EN}(0)}(\rho_{\mathrm{A}},\rho_{\mathrm{B}}) = -\frac{Z_{\mathrm{B}}c_{\mathrm{A}}\mathrm{erf}(\sqrt{\lambda_{\mathrm{A}}}\|r_{\mathrm{A}} - r_{\mathrm{B}}\|)}{\|r_{\mathrm{A}} - r_{\mathrm{B}}\|} - \frac{Z_{\mathrm{A}}c_{\mathrm{B}}\mathrm{erf}(\sqrt{\lambda_{\mathrm{B}}}\|r_{\mathrm{A}} - r_{\mathrm{B}}\|)}{\|r_{\mathrm{A}} - r_{\mathrm{B}}\|} \tag{S7}$$

$$\mathrm{XC}_{\mathrm{AB}}^{\mathrm{EN}(1)}(\rho_{\mathrm{A}},\rho_{\mathrm{B}}) = -4\pi\big(Z_{\mathrm{B}}\rho_{\mathrm{A}}(r_{\mathrm{B}}) + Z_{\mathrm{A}}\rho_{\mathrm{B}}(r_{\mathrm{A}})\big) \tag{S8}$$

$$\mathrm{XC}_{\mathrm{AB}}^{\mathrm{EN}(2)} = -\frac{8Z_{\mathrm{B}}c_{\mathrm{A}}\lambda_{\mathrm{A}}{}^{5/2}(2\lambda_{\mathrm{A}}\|r_A - r_{\mathrm{B}}\|^2 - 3)}{\sqrt{\pi}\exp(\lambda_{\mathrm{A}}\|r_{\mathrm{A}} - r_{\mathrm{B}}\|^2)} - \frac{8Z_{\mathrm{A}}c_{\mathrm{B}}\lambda_{\mathrm{B}}{}^{5/2}(2\lambda_{\mathrm{B}}\|r_{\mathrm{A}} - r_{\mathrm{B}}\|^2 - 3)}{\sqrt{\pi}\exp(\lambda_{\mathrm{B}}\|r_{\mathrm{A}} - r_{\mathrm{B}}\|^2)}, \tag{S9}$$

higher order terms for the electron-electron and electron-nuclei exchange and correlation functionals can be easily calculated using the following recursive formulas:

$$\mathrm{XC}_{\mathrm{AB}}^{\mathrm{EE}(n)} = \frac{1}{d^2}\frac{\partial}{\partial d}\left(d^2\frac{\partial}{\partial d}\mathrm{XC}_{\mathrm{AB}}^{\mathrm{EE}(n-1)}\right) \tag{S10a}$$

$$\mathrm{XC}_{\mathrm{AB}}^{\mathrm{EN}(n)} = \frac{1}{d^2}\frac{\partial}{\partial d}\left(d^2\frac{\partial}{\partial d}\mathrm{XC}_{\mathrm{AB}}^{\mathrm{EN}(n-1)}\right), \tag{S10b}$$

where $d = \|r_{\mathrm{A}} - r_{\mathrm{B}}\|$.

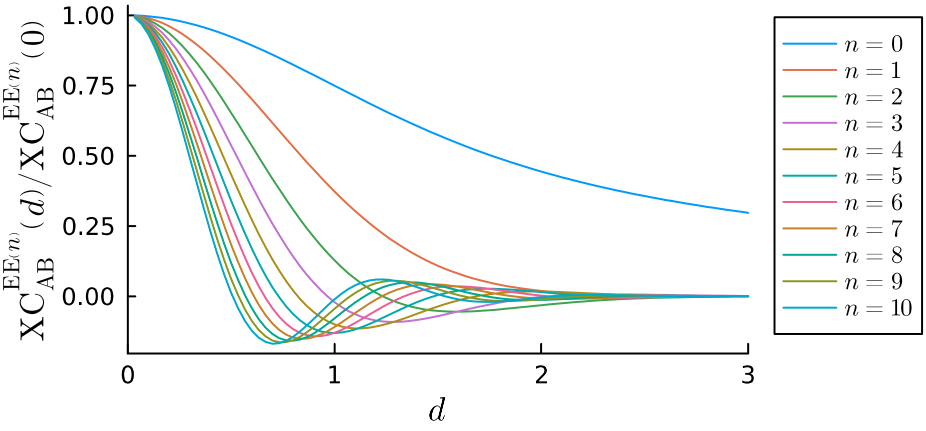

Figure S1 *Plotted examples of first ten normalized XC electron-electron functionals of a fictitious system of two atoms whose electron clouds are given by just one s-type Gaussian function. The plots are traced as functions of the interatomic distance $d = \|\lambda_A - \lambda_B\|$, and for this example $\lambda_A = 2.45$ and $\lambda_B = 1.65$.*

Table S3 *Multiplying coefficients in the summation of the exchange and correlation functionals obtained from least squares minimization against the reference ab initio water dimer data for the full electron and ECP models.*

k	Full Electron Fit		ECP Fit	
	a_k	b_k	a_k	b_k
0	-1.2626×10^{-2}	2.5106×10^{-2}	-2.0439×10^{-3}	3.9926×10^{-3}
1	-1.5394×10^{-1}	2.0293×10^{-1}	8.5734×10^{-1}	-14.886×10^{-1}
2	-8.0226×10^{-2}	-5.7248×10^{-2}	3.6408×10^{-3}	1.9678×10^{-1}
3	-1.6789×10^{-3}	-8.7052×10^{-3}	9.2417×10^{-3}	5.6710×10^{-3}
4	-1.0836×10^{-4}	-3.6683×10^{-4}	8.7046×10^{-4}	-1.1361×10^{-3}
5			1.3776×10^{-5}	-1.2207×10^{-4}
6			6.1397×10^{-7}	-1.2274×10^{-5}
